# PPARδ Attenuates Alcohol-Mediated Insulin Resistance by Enhancing Fatty Acid-Induced Mitochondrial Uncoupling and Antioxidant Defense in Skeletal Muscle

**DOI:** 10.3389/fphys.2020.00749

**Published:** 2020-07-14

**Authors:** Jin-Ho Koh, Ki-Hoon Kim, Sol-Yi Park, Yong-Woon Kim, Jong-Yeon Kim

**Affiliations:** Department of Physiology, College of Medicine, Yeungnam University, Daegu, South Korea

**Keywords:** alcohol, PPARδ, UCP3, carnitine-acylcarnitine translocase, mitochondrial uncoupling, H_2_O_2_, antioxidant defense, fatty acid

## Abstract

Alcohol consumption leads to the dysfunction of multiple organs including liver, heart, and skeletal muscle. Alcohol effects on insulin resistance in liver are well evidenced, whereas its effects in skeletal muscle remain controversial. Emerging evidence indicates that alcohol promotes adipose tissue dysfunction, which may induce organ dysregulation. We show that consumption of ethanol (EtOH) reduces the activation of 5′AMP-activated protein kinase (AMPK) and mammalian target of rapamycin (mTOR) as well as the protein of carnitine palmitoyltransferase 1 (CPT1) and glucose transporter type 4 (GLUT4) in C_2_C_12_ myotube. We observed that chronic EtOH consumption increases free fatty acid levels in plasma and triglyceride (TG) accumulation in skeletal muscle and that these increases induce insulin resistance and decrease glucose uptake. Hence, ethanol dysregulates metabolic factors and induces TG accumulation. We found peroxisome proliferator-activated receptor β/δ (PPARδ) activation recovers AMPK activation and increases carnitine-acylcarnitine translocase (CACT) protein. These effects may contribute to enhance mitochondrial activation via uncoupling protein 3 (UCP3) when fatty acids are used as a substrate, thus reduces EtOH-induced increases in TG levels in skeletal muscle. In addition, PPARδ activation recovered EtOH-induced loss of protein kinase B (AKT) phosphorylation at serine 473 via rapamycin-insensitive companion of mammalian target of rapamycin (Rictor) activation. Importantly, PPARδ activation enhanced mitochondrial uncoupling via UCP3. Taken together, the study shows PPARδ enhances fatty acid utilization and uncoupled respiration via UCP3 and protects against EtOH-induced lipotoxicity and insulin resistance in skeletal muscle.

## Introduction

Body metabolic homeostasis is tightly regulated by the liver-adipose tissue-skeletal muscle axis, which importantly regulates the storage, synthesis, and consumption of energy substrates, and thus, disruption of this axis may cause various metabolic diseases. White adipose tissue (WAT) plays roles in lipid storage and release according to supply and demand. However, it has been shown that alcohol abuse disrupts these functions (Zhong et al., 2012) and causes the developments of liver diseases such as steatosis, steatohepatitis, and fibrosis (Gao and Bataller, 2011; Zhang et al., 2017). Skeletal muscle is a primary site for glucose disposal and storage, and insulin resistance in muscle is fundamental to the metabolic dysregulations associated with obesity and physical inactivity. It is well known that excessed fatty acid accumulation in peripheral tissue with high metabolic active may cause metabolic dysregulation of glucose, known as insulin resistance due to glucose fatty-acid cycle, and the previous study has shown that glucose transporter type 4 (GLUT4), a rate-limiting factor for glucose uptake, in mice skeletal muscle is decreased by long-term high-fat diet (Koh et al., 2019b). Hence, it is possible alcohol abuse causes insulin resistance in skeletal muscle by disrupting lipid homeostasis in adipose tissues. However, it is not known how metabolic dysregulation of the skeletal muscle-adipose tissue axis caused by chronic alcohol abuse induces insulin resistance and metabolic disorder in skeletal muscle.

It has been shown that excessive alcohol consumption disrupts the regulation of protein synthesis, which may induce muscle atrophy and lower contents of metabolic enzyme. Mammalian target of rapamycin (mTOR) is signaling factor for protein synthesis, lipid, and glucose metabolism (Mao and Zhang, 2018). Since previous studies have shown that alcohol abuse decrease mTOR pathway, insulin signaling factors and various enzymes for glucose uptake and fatty acid oxidation could be reduced in skeletal muscle. Furthermore, incomplete β-oxidation via lower enzyme and high reactive oxygen species (ROS) via mitochondrial dysfunction induced by exceed fatty acid accumulation is also one of the cause to lower glucose uptake via insulin resistance (Koh et al., 2019b). Indeed, excessive H_2_O_2_ release from mitochondria caused by a high-fat diet results in insulin resistance (Anderson et al., 2009). Therefore, metabolic dysregulation of adipose tissue induced by long-term alcohol consumption may downregulate glucose uptake in skeletal muscle by lowering insulin sensitivity via various pathways including mitochondrial dysfunction and increased ROS.

Peroxisome proliferator-activated receptors (PPARs) are a family of nuclear receptors and transcription factor; three isoforms (α, β/δ, or γ) are expressed in skeletal muscle. Since PPARβ/δ (PPARδ) is the predominant isoform that regulates fatty acid oxidation (Dressel et al., 2003; Tanaka et al., 2003; Wang et al., 2003) and that is essential to maintain a normal level of mitochondria (Koh et al., 2017) and GLUT4 in skeletal muscle (Koh et al., 2019a), we speculated that PPARδ is one of the candidates to improve the metabolism in skeletal muscle against lipid dysregulation due to alcohol abuse. The previous study has shown that PPARδ can be activated by GW501516 (GW; a PPAR*δ* receptor agonist) that a key feature is the induction of skeletal muscle glucose and fatty acid metabolism as well as antioxidant expression (Fan et al., 2017).

Hence, we sought to determine whether GW treatment in mice or C_2_C_12_ myotubes can increase fatty acid utilization and mitochondrial respiration, and prevent ethanol (EtOH)-induced insulin resistance in muscle of mice. We further investigated whether various PPAR*δ*-influenced molecular pathways affect insulin sensitivity and oxidative stress in skeletal muscle. The mechanistic insights provided offer potential therapeutic approaches to counteract insulin resistance in skeletal muscle induced by alcohol abuse.

## Materials and Methods

### Animal Study

All institutional and governmental regulations regarding the ethical use of were complied with during this study, which was approved by the Institutional Animal Care and Use Committee of Yeungnam University (YUMC-AEC2018-034). Male ICR mice weighing ∼28 g and C57BL6 mice weighing ∼20 g were purchased form Koatech (Gyeonggi, South Korea), ICR mice were used to analyze lipid profile and histochemistry in adipose tissue, and C57BL6 mice were used to determine circulating glucose disposal and molecular phenotype in skeletal muscle. Mice were housed under a 12:12-h light/dark cycle and provided a chow diet and water *ad libitum*. GW501516 (GW) was administered in diet at 40 mg/kg, and 5% EtOH was administered *ad libitum* for 5 weeks.

### Cell Lines

C_2_C_12_ mouse cells were purchased from ATCC (cat. no. CRL-1772; Manassas, VA, United States), and cell study was performed as previously described (Koh et al., 2019a). Briefly, cells were maintained in 5% CO_2_ at 37°C and grown in DMEM (Sigma-Aldrich, St. Louis, MO, United States) containing 10% fetal bovine serum (Sigma-Aldrich, St. Louis, MO, United States), penicillin (100 U/ml), and streptomycin (100 μg/ml) (Thermo Fisher Scientific, Waltham, MA, United States). C_2_C_12_ cells were differentiated into myotubes by changing the medium to 2% horse serum (Thermo Fisher, Waltham, MA, United States) containing penicillin (100 U/ml) and streptomycin (100 μg/ml) (Thermo Fisher, Waltham, MA, United States); 50 mM or 30 mM of EtOH was treated in myotubes.

### Plasmid Transfection

We used pcDNA 3.1-Myc-PPAR*δ* vector, which was constructed as previously described (Koh et al., 2019a). HyPer-mito (Evrogen, Moscow, Russia), which is a sensor to detect mitochondrial hydrogen peroxide (H_2_O_2_), is a fluorescent protein and was gifted by Dr. Dong-Hyung Cho at Kyungpook National University, South Korea. pcDNA 3.1-empty vector and Myc-PPAR*δ* vector were transfected using Lipofectamine 2000 (Thermo Fisher, Waltham, MA, United States).

### Plasma Free Fatty Acid and Triglyceride Concentrations

Plasma free fatty acid and triglyceride concentrations were measured using a kit obtained from Waco Chemicals (Richmond, VA, United States).

### Adipose Tissue Staining

Adipose tissues were fixed with 10% formalin solution, embedded in paraffin blocks, and sectioned at 4 μm. Hematoxylin and eosin and Masson’s trichrome staining were performed using standard methods. Digital images were captured using an Aperio CS digital pathology slide scanner (Leica, Germany). Adipocyte size was analyzed using the ImageJ program (NIH, Bethesda, MD, United States).

### Intraperitoneal Glucose Tolerance Testing (IPGTT)

After 5 weeks of EtOH with or without GW or saline treatment, mice were fasted overnight, and blood samples were collected from a tail vein to determine basal blood glucose levels. We performed IPGTT as previously described (Shin et al., 2019). Briefly, glucose (1.5 g/kg body weight) was injected intraperitoneally, and blood samples were collected at 15, 30, 60, 90, and 120 min. Blood glucose levels were measured using OneTouch blood glucose meters (LifeScan Europe, Switzerland).

### Triglyceride Measurement

Muscle triglyceride concentrations were determined by extracting total lipids from clamp-frozen muscle samples using chloroform-methanol (2:1 vol/vol), as described by Folch et al. (1957). After separating chloroform and methanol-water phases and phospholipid removal, samples were processed using Frayn and Maycock’s modification (Frayn and Maycock, 1980) of the Denton and Randle method (Denton and Randle, 1967). Triglycerides were quantified spectrophotometrically using an enzymatic assay kit (Waco Chemicals, Richmond, VA, United States).

### 2DG-Glucose Uptake

Glucose uptake by myotubes was determined using ^14^C-2-deoxy-D-glucose (2DG, PerkinElmer Life and Analytical Sciences, United States). The glucose uptake method described below has been reproduced in part from the previous study (Shin et al., 2019). Briefly, myotubes were fasted for 2 h in serum-free medium, treated with insulin (100 nM) for 1 h, ^14^C-2DG was then added to each well, and 20 mM cytochalasin B was added to control cells. Cells were lysed with 60 mM NaOH and insulin-stimulated myotube glucose uptakes were determined using a Liquid Scintillation Analyzer (PerkinElmer).

### Insulin Sensitivity

Myotubes were fasted for 2 h in serum-free medium containing insulin (100 nM) for 1 h and then harvested to evaluate AKT activation.

### Western Blot Analysis

Western blots were performed as previously described (Koh et al., 2019a). Briefly, frozen tissues were powdered and then homogenized at 15:1 (vol/wt) in ice-cold RIPA buffer supplemented with protease inhibitor cocktail (Thermo Fisher Scientific, Houston, TX, United States). Protein contents were measured using a DC-protein assay kit (Bio-Rad). The antibodies used were as follows: AMPK (cat. no. 2793), phospho-AMPK (pAMPK; cat. no. 2541), mammalian target of rapamycin (mTOR; cat. no. 2972), phosphor-mTOR (p-mTOR; cat. no. 2971), regulatory associated protein of mTOR (Raptor; cat no. 2280), phosphor-Ser792-Raptor (p-Raptor; cat. no. 2083), rapamycin-insensitive companion of mammalian target of rapamycin (Rictor; cat. no. 2114), phosphor-Thr1135-Rictor (p-Rictor; cat. no. 3806), phosphor-Thr308-Akt (pT308-Akt; cat. no. 13038), phosphor-Ser473-Akt (pS473-Akt; cat. no. 4060), and Akt (cat. no. 2920) from Cell Signaling Technologies (Danvers, MA, United States); all Cell Signaling antibody were diluted 1:1,000. Superoxide dismutase 2 (SOD2; cat. no. sc-137254. 1:500 dilution) and carnitine palmitoyltransferase (CPT1; cat. no. 393070, 1:1,000 dilution) were from Santa Cruz Biotechnology. β-actin (cat. no. A5441, 1:1,000 dilution) was from Sigma Aldrich (St. Louis, MO, United States). Long-chain acyl-CoA dehydrogenase (LCAD; cat. no. ab196655, 1:1,000 dilution) and glucose transporter type 4 (GLUT4; cat. no. ab-654, 1:1,000 dilution) were from Abcam (Cambridge, MA, United States). Citrate synthase (CS; cat. no. CISY11-A, 1:5,000 dilution) was from Alpha Diagnostics (San Antonio, TX, United States). ATP synthase (cat. no. 459240, 1:5,000 dilution), succinate ubiquinone oxidoreductase (SUO; cat. no. 459200, 1:5,000 dilution), and NADH ubiquinone oxidoreductase (NUO; cat. no. 459210, 1:5,000 dilution) were from Thermo Fisher Scientific (Waltham, MA, United States). Uncoupling protein 3 (UCP3; cat. no. 10750-AP, 1:1,000 dilution) and carnitine-acylcarnitine translocase (CACT; cat. no. 19363-AP, 1:1,000 dilution) were from Proteintech (Rosemont, IL, United States).

### Hydrogen Peroxide Emission Analysis

We performed hydrogen peroxide emission analysis as previously published (Koh et al., 2019b). Briefly, palmitate or saline treated PPAR*δ* or empty vector (EV) myotubes in 75 t flasks were washed with DPBS and suspended in 0.25% trypsin-EDTA medium. Myotubes were then centrifuged, re-suspended in respiration medium [105 mM K-MES, 30 mM KCL, 10 mM KH2PO4, 5 mM MgCl2-6H2O, 5 mg/ml BSA] pH 7.4 supplemented with 1 mM EGTA (pH 7.3)] including 10 mM Amplex Red, horseradish peroxidase 1 U/ul, superoxide dismutase 10 U/ul, and treated with 500 μg/ml digitonin (∼50% TLC, Sigma, St. Louis, United States) to permeabilize membranes. Glutamate (5 mM, 2 mmol/L) and malate (1 mmol/L) were then added to stimulate H_2_O_2_ production under state 4 respiration conditions. A fluorolog 3 (Horiba Jobin Yvon, Edison, Piscataway, NJ, United States) spectrofluorometer was used to monitor Amplex Red (Invitrogen, Carlsbad, United States) oxidation in myotubes.

### Mitochondria Isolation

Fresh tissues were homogenized in first isolation buffer containing 10 mM EDTA, 215 mM D-mannitol, 75 mM sucrose, 20 mM HEPES, and 0.1% free-fatty acid BSA (pH 7.4) using Potter-Elvehjem tissue homogenizer and then centrifuged at 700 *g* for 10 min at 4°C followed by transfer supernatant to new microcentrifuge tube. The supernatant was centrifuged at 10,500 *g* for 10 min, then supernatants were discarded and pellets (mitochondria) were resuspended in 100 μl of second isolation buffer [3 mM EDTA, 215 mM D-mannitol, 75 mM sucrose, 20 mM HEPES, and 0.1% free-fatty acid BSA (pH 7.4)].

### Mitochondria Oxygen Consumption

Mitochondrial oxygen consumption was assessed using an Oxytherm System (Hansatech Instruments), as previously described (Finlin et al., 2018). Briefly, mitochondria were added to the Oxytherm chamber containing 500 μl of mitochondrial respiration buffer (125 mM KCl, 2 mM MgCl_2_, 2.5 mM KH_2_PO_4_, 20 mM HEPES, pH adjusted to 7.2) at 37°C under constant stirring. Complex I-driven respiration was initiated by adding pyruvate/malate (5 mM and 2.5 mM, respectively) or palmitoyl-carnitine/malate (5 mM and 2.5 mM, respectively) followed by ADP (to 300 μM in 2 steps) to induce State 3 respiration. ATP synthase activity was then inhibited by adding 2.5 μM oligomycin to induce State 4 respiration. Finally, maximum respiration was measured in the presence of 10 μM trifluoromethoxy carbonylcyanide phenylhydrazone (FCCP). OCRs were determined and expressed as nanomoles/min. Mitochondrial respiratory uncoupling via UCP3 was calculated as the difference between State 3 respiration (with FFA as substrate) and State 4 respiration (induced by oligomycin).

## Results

### EtOH Increased Lipolysis in Adipose Tissue, Induced Triglyceride (TG) Accumulation, and Dysregulated Fatty Acid Oxidation in Skeletal Muscle

**FIGURE 1:**
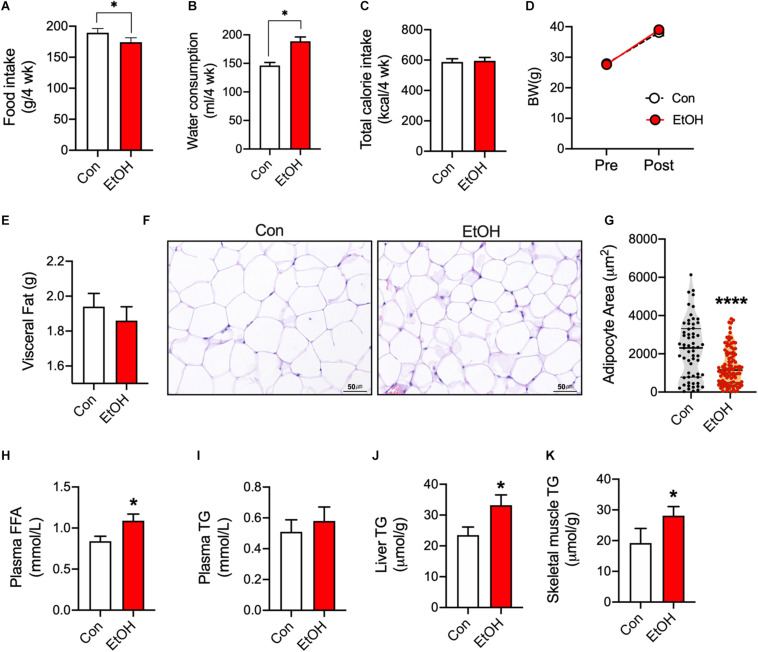
Chronic alcohol consumption released fatty acids from adipose tissue and increased triglyceride accumulation in mouse skeletal muscle. **(A)** Food intake (*n* = 6 mice), **(B)** Water consumption (*n* = 6 mice), and **(C)** Total calorie intake (*n* = 6). Values are means ± SE. *P < 0.05. Significance was determined using the student’s *t*-test. **(D)** 5% EtOH consumption did not affect body weights (*n* = 6 animal/group). **(E)** Visceral fat weights (*n* = 6 tissue/group). **(F)** Sections of epididymal white adipose tissue from mice after treatment with water or 5% EtOH. **(G)** Adipocyte area was quantified from section of adipose tissue. Values are means ± SE. ****P < 0.0001. Significance was determined using the student’s *t*-test. **(H)** EtOH consumption increased plasma free fatty acid levels (FFA) (*n* = 6 plasma/group). Values are means ± SE. *P < 0.05. Significance was determined using the student’s *t*-test. **(I)** Plasma triglyceride (TG) level (*n* = 6 plasma samples/group). **(J**,**K)** EtOH consumption increased liver and skeletal muscle TG levels (*n* = 6 tissue samples/group). Values are means ± SE. *P < 0.05 versus non-treated controls. Significance was determined using student’s *t*-test. **(G)** H&E staining was used to determine adipocyte sizes at different EtOH concentrations.

To test the effects of chronic alcohol consumption, mice consumed 5% EtOH for 4 weeks. Food intake was lower in EtOH consumption mice than control (Figure 1A), but water consumption was higher (min–max; 138–152 per 4 weeks) in 5% EtOH consumption group than control (min–max; 174–195 per 4 weeks) (Figure 1B). EtOH treatment did not changes body weights (BW, Figure 1D) because no difference of total calorie intake (Figure 1C). EtOH consumption slightly decreases visceral fat weight (Figure 1E) but significantly increased plasma free fatty acid (FFA) levels as compared with controls (Figure 1H). Since the source the increased FFA observed in plasma was adipose tissue, we examined epididymal adipocyte sizes by hematoxylin-eosin staining. Representative sections showed that area of adipocytes in 5% EtOH consumption mice were significantly smaller than in controls (Figures 1F,G). We also found EtOH consumption increased the activation of hormone sensitive lipase in rats (Supplementary Figure S1). Furthermore, we observed that triglyceride (TG) levels in mouse skeletal muscle (Figure 1K) and liver (Figure 1J) were higher than in controls, but not plasma TG (Figure 1I). These results show 5% EtOH consumption induced the accumulation of TG in skeletal muscle and that this accumulation might be associated with elevated FFA level resulted from increased lipolysis in adipose tissue.

### EtOH Downregulated Metabolic Enzymes and Signaling Factor in C_2_C_12_ Myotubes

**FIGURE 2:**
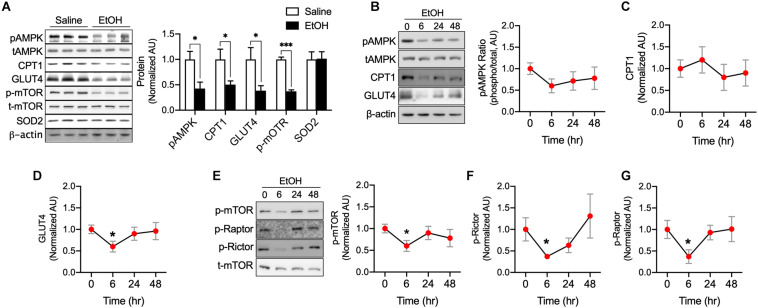
EtOH directly downregulated pAMPK, CPT1, GLUT4, and p-mTOR content in myotubes. **(A)** Protein content of the key metabolic factors, AMPK, CPT1, GLUT4, and mTOR, and of the antioxidant SOD2 was measured in myotubes exposed to 50 mM EtOH for 24 h (*n* = 6 myotubes/group). Values are means ± SE. *P < 0.05 versus non-treated controls. Significance was determined using the student’s *t*-test. **(B–G)** Protein content of AMPK, CPT1, GLUT4, mTOR, Raptor, and Rictor in myotubes was measured after exposure to 30 mM EtOH for different times (*n* = 6 myotubes/time point). Values are means ± SE. *P < 0.05 and ***p < 0.001 versus baseline. Significance was determined using one-way ANOVA.

We next studied whether EtOH directly influences various metabolic enzymes and signaling factors related to fatty acid and glucose metabolism using C_2_C_12_ myotubes. Western blotting was used to assess AMPK, mTOR, CPT1, GLUT4, and SOD2 protein. It has been documented that AMPK, a cellular energy sensor, clearly link to muscle glucose uptake (Koh et al., 2019a) and metabolism (Gan et al., 2011) as well as fatty acid oxidation and mitochondrial biogenesis (O’Neill et al., 2013). We found that AMPK is significantly decreased by 50 mM EtOH in myotubes. Muscle-specific CPT1 knockout mice have been shown to exhibit low levels of fatty acid oxidation and increased lipid accumulation in the muscle (Wicks et al., 2015), these exceed fatty acid accumulation results in a decrease in GLUT4 content (Koh et al., 2019b), as expected, CPT1 and GLUT4 in myotubes were decreased by 50 mM EtOH treatment (Figure 2A). However, since the myotubes were incubated with high glucose, fatty acid may not be accumulated much in the myotubes by EtOH treatment, and thus, it appears that GLUT4 protein synthesis was inhibited by lack of mTOR signaling induced by 50 mM EtOH (Figure 2A), furthermore, ROS can also affect the protein degradation (Kocha et al., 1997), therefore, it is possible that 50 mM EtOH may decrease GLUT4 content via lack of mTOR signaling and/or ROS. Cellular energy metabolism is tightly regulated by substrate concentrations and signaling intensity, and thus, we studied the temporal effects of a lower concentration of EtOH (30 mM). We observed that this lower concentration slightly decreased the phosphorylation of AMPK. However, GLUT4, phosphorylated mTOR, Raptor, and Rictor amount in myotubes exposed to this lower concentration were significantly diminished after 6 h of treatment and then gradually recovered (Figures 2B–G), presumably because almost all of the ethanol appeared to have been metabolized at 48 h. These results suggest EtOH toxicity can directly downregulate signaling related to energy metabolism and protein synthesis even lower amount EtOH (Figure 2).

### PPARδ Restored Lower Glucose Disposal Rate Induced by EtOH via mTORC2/S473-AKT and GLUT4

**FIGURE 3:**
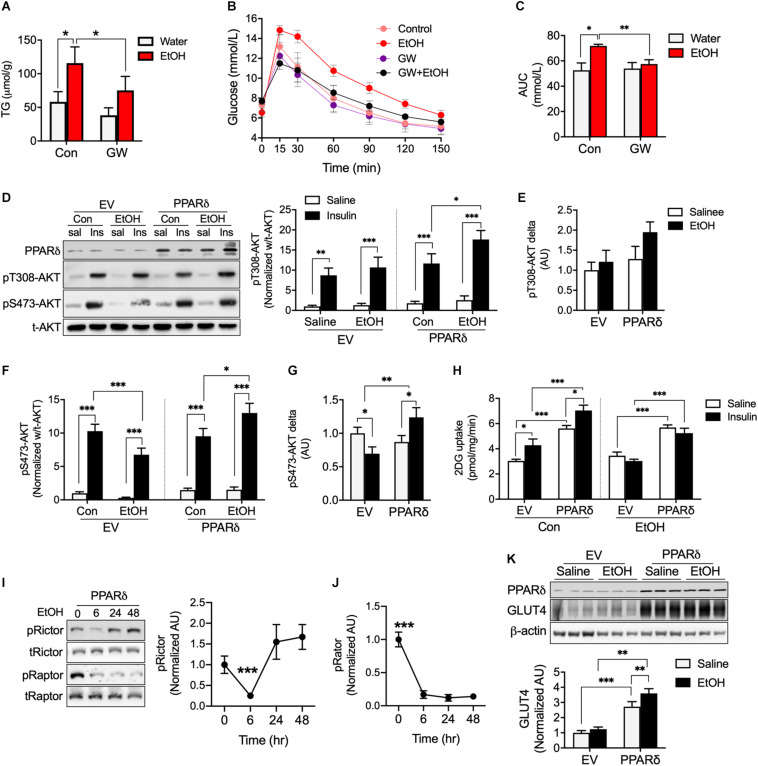
PPARδ activation attenuated EtOH-induced fat accumulation and insulin resistance in mouse skeletal muscle. **(A)** TG levels in skeletal muscle of GW, GW plus EtOH, EtOH consumed mice (*n* = 6 muscles/group). **(B**,**C)** Blood glucose disposal capacities were measured using the intra-peritoneal glucose tolerance test (IPGTT) (*n* = 6/group). **(D–H)** AKT signaling and glucose uptakes were measured in EV or PPARδ overexpressing myotubes treated with saline or EtOH (*n* = 6 myotubes/group). **(I**,**J)** Activation of Rictor and Raptor was measured time dependently in EtOH and PPARδ overexpressing myotubes (*n* = 6 myotubes/group). **(K)** GLUT4 was measured in EV or PPARδ overexpressing myotubes treated with saline or EtOH (*n* = 6 myotubes/group). Values are means ± SE. Significance was determined using two-way ANOVA with Tukey’s correction. *p < 0.05, **P < 0.01, and ***P < 0.001.

PPAR*δ* activation has shown to increase fatty acid oxidation (Fan et al., 2017) and PPAR*δ* overexpression has been reported to increase glucose uptake (Koh et al., 2019a). Since acute 5% EtOH consumption reduces metabolic regulation factors related to PPAR*δ* including pAMPK, CPT1, and GLUT4 (Figure 2), we speculated that PPAR*δ* can directly or indirectly involved in metabolic disorder by chronic alcohol consumption. We observed mice treated with 5% EtOH for 5 weeks had significantly higher TG levels in muscle tissue than controls, but GW plus 5% EtOH treated mice had lower TG levels than 5% EtOH treated mice (Figure 3A). IPGTT results showed the circulating glucose disposal rate in 5% EtOH treated mice was lower than in non-treated controls, but that co-treatment with GW maintained glucose disposal rates in 5% EtOH treated mice at the non-treated control level (Figures 3B,C). Since skeletal muscle responsible for approximately 80% of insulin-mediated glucose uptake in the postprandial state (Thiebaud et al., 1982), we tested insulin sensitivity in mouse muscle cells according to the treatment of EtOH and GW (Figures 3D–G). We found that glucose uptake induced by insulin in EtOH 30 mM treated myotubes was significantly lower than in saline treated myotubes (Figure 3H). However, PPAR*δ* overexpression in myotubes significantly increased glucose uptake level in both of insulin dependent and independent manner (Figure 3H). To identify whether the mechanism responsible for this glucose uptake was regulated by EtOH or PPAR*δ*, we examined AKT signaling induced by insulin. It was found 30 mM EtOH did not interrupt insulin-induced threonine 308 AKT (t308-AKT) signaling, and that insulin-induced increases in t308-AKT occurred in PPAR*δ* overexpressing myotubes (Figures 3D,E). In contrast, serine 473 AKT (S473-AKT) phosphorylation was inhibited by EtOH, and this effect was reversed by PPAR*δ* (Figures 3F,G). We found PPAR*δ* accelerated the normalization of Rictor (Figure 3I) but not Raptor activation in EtOH exposed-myotubes (Figure 3J).

We also observed that EtOH did not decrease GLUT4 protein content after 24 h of treatment of EtOH same as Figure 2D; however, PPAR*δ* overexpression in myotubes increased GLUT4 protein amount regardless of EtOH and saline treatment (Figure 3K). Together, these results suggest the effects of PPAR*δ* including the restoration of insulin mediated-lower S473-AKT signaling and increase GLUT4 protein content may affect insulin-stimulated glucose disposal rate in blood.

### PPARδ Activation Induced Mitochondrial Respiration Uncoupling in Skeletal Muscle

**FIGURE 4:**
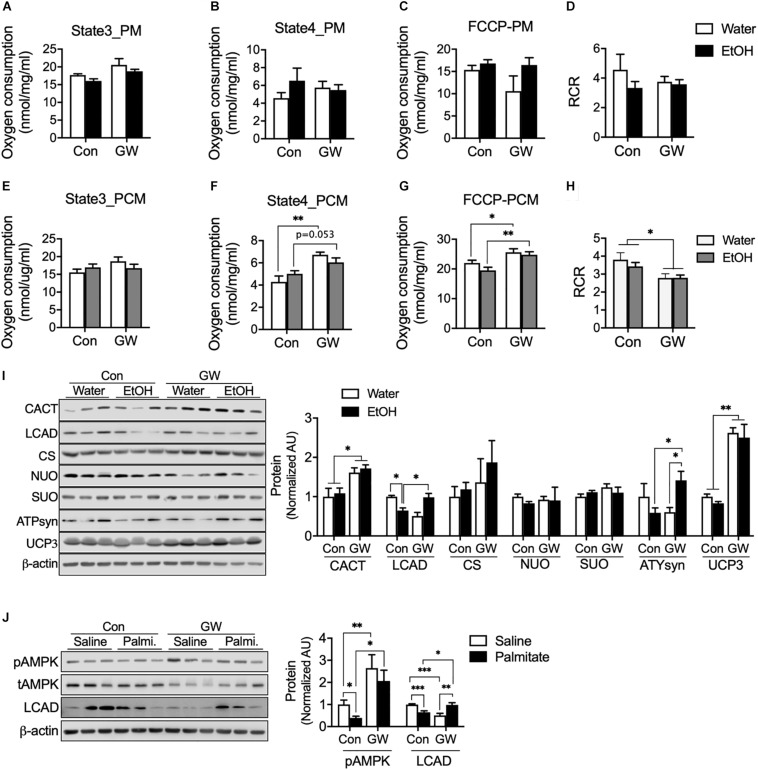
PPARδ increased mitochondrial uncoupling via UCP3 when fatty acids were used as substrates. **(A–H)** Oxygen consumption was measured in isolated mitochondria from skeletal muscle using various substrates (*n* = 6–7 muscles/group). PM, pyruvate and malate; PCM, palmitoyl-carnitine and malate. **(I)** Protein **content** of mitochondrial enzymes (CACT, LCAD, CS, NUO, SUO, ATPsyn, UCP3) was measured in the skeletal muscles of GW, EtOH, or GW plus EtOH treated mice (*n* = 6–7 muscles/group). **(J)** AMPK activation and LCAD protein content were measured in myotubes treated with GW and/or palmitate. Noncontiguous gel lanes are clearly demarcated by black line. Values are means ± SE. Significance was determined by two-way ANOVA with Tukey’s correction. *P < 0.05, **P < 0.01, and ***P < 0.001.

We were curious that PPAR*δ* activation can induce modification of the mitochondrial respiration chain to protect cellular against metabolic disorder induced by EtOH. We tested oxygen consumption rates in mitochondria extracted from the muscles of EtOH and/or GW treated mice, and found neither EtOH nor GW influenced mitochondrial respiration (Figures 4A–C) or respiration control ratio [RCR, indicator of the coupling state of mitochondria (Rhein et al., 2010)] (Figure 4D) when pyruvate and malate (PM) was used as a substrate. However, state 4 (Figure 4F) and maximal oxygen respiration (Figure 4G) of mitochondria from EtOH plus GW and from GW treated mice were higher than in non-treated controls when palmitoyl-carnitine and malate (PCM) was used as substrate. We also found coupling efficiency in mitochondria from GW treated mice was lower than in non-treated control mice when PCM was used as substrate (Figure 4H). Next, we sought to determine potential mechanisms by which GW influences mitochondrial respiration. Contrary to that observed in the cell study, AMPK activation and CACT protein content were not down-regulated by EtOH, presumably because we isolated muscles 36 h after alcohol treatment to eliminate the acute effects of alcohol. CACT transports acylcarnitine derived from fatty acid oxidation into the mitochondrial matrix (Ogawa et al., 2000), and the previous study has shown that CACT deficiency dysregulates fatty acid oxidation (Pande et al., 1993), in the present study, CACT protein content in mouse muscle was unaffected by EtOH treatment, but GW increased CACT protein amount when administered with water or EtOH (Figure 4I). LCAD is a key enzyme in mitochondrial β-oxidation (Lea et al., 2000), and we observed LCAD protein content were lower in the muscles of EtOH treated mice than in non-treated controls, but not in EtOH plus GW treated mice (Figure 4I). Since GW consumption is known to increase fatty acid oxidation (Fan et al., 2017), we did not expect this result, and thus, we investigated the effect of GW treatment with or without palmitate on AMPK activation and LCAD protein content in myotubes. We found that palmitate down-regulates AMPK phosphorylation and LCAD protein content, but GW increases AMPK activation regardless of palmitate presence, and LCAD content was recovered by GW with palmitate (Figure 4J). These results suggest GW activation is regulated in a fatty acid concentration-dependent manner, and thus, GW may not be activated much in the low or normal level of TG concentration in the tissue (Figures 3A, 4I). No differences in citrate synthase (CS; involved in the tricarboxylic acid (TCA) cycle), NU), or SUO protein content were observed in skeletal muscle (Figure 4I). ATPsyn amount was slightly lower in 5% EtOH treated mouse muscle than in non-treated controls, but identical in EtOH plus GW treated mice and non-treated controls (Figure 4I). Furthermore, GW significantly increased uncoupling protein 3 (UCP3) levels when treated alone or with EtOH (Figure 4I). Collectively, GW appears to induce mitochondrial respiration uncoupling via UCP3 and to enhance fatty acid oxidation in mitochondria when fatty acids are used as substrates (Figure 4).

### PPARδ Activation Lowered ROS Emission in Myotubes

**FIGURE 5:**
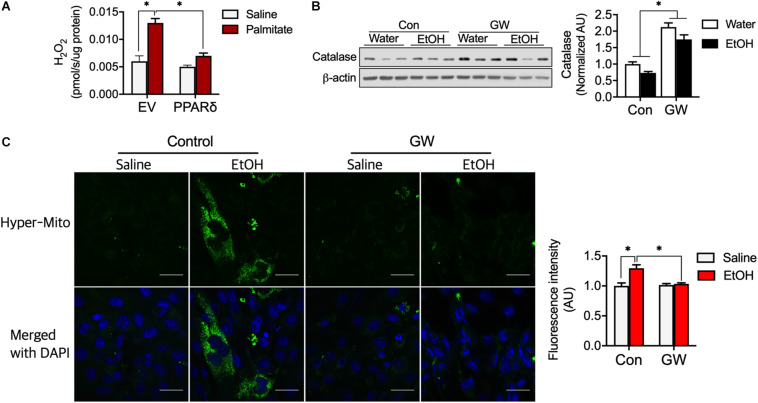
PPARδ attenuated fatty acid-induced ROS by increasing catalase protein content. **(A)** PPARδ attenuated palmitate increased H_2_O_2_ levels in myotubes (*n* = 6 myotubes/group). **(B)** PPARδ activation increased catalase protein content in mouse skeletal muscles (*n* = 6–7 muscles/group). **(C)** Representative image of H_2_O_2_ emission from mitochondria obtained using an H_2_O_2_ sensor (HyPer-mito, green, Moscow). Nuclei were stained with DAPI. Scale bar, 30 μm. Values are means ± SE. Significance was determined by two-way ANOVA with Tukey’s correction, *P < 0.05.

Reactive oxygen species emissions from mitochondria can induce insulin resistance (Anderson et al., 2009). We determined H_2_O_2_ emission from myotubes and found palmitate increased H_2_O_2_ emission (Figure 5A), but that PPARδ blocked H_2_O_2_ emission induced by palmitate (Figure 5A). We also observed that PPARδ activation increased catalase protein content in skeletal muscle of both water and EtOH consumed mice (Figure 5B). To test whether PPARδ activation by GW can reduce H_2_O_2_, a plasmid of mitochondria H_2_O_2_ sensor (HyPer-mito) was transfected into C_2_C_12_ muscle cells. We found EtOH increased HyPer-mito fluorescence intensity and that this was recovered by GW (Figure 5C). These data indicate that PPARδ protects muscles against both palmitate and EtOH induced ROS emission via catalase.

## Discussion

PPAR*δ* is a critical regulator of energy metabolism in skeletal muscle, and is highly sensitive to energetic challenges such as those caused by exercise (Koh et al., 2019a) and fasting (Gaudel and Grimaldi, 2007). Studies on the effects of alcohol have focused on liver function and diseases (Louvet and Mathurin, 2015), but not on skeletal muscle, which is primarily responsible for whole-body energy metabolism. Here, we suggested PPAR*δ* can improve EtOH-induced lowered blood glucose disposal rate by enhanced mitochondrial respiration with fatty acid and antioxidant defense in skeletal muscle.

Direct exposure of myotubes to ethanol reduces in the metabolic regulator such as GLUT4, CPT1, and activation of AMPK and mTOR. The present study shows that ethanol time or dose-dependently reduce the mTOR signaling, and which clearly affects protein synthesis, lipid and glucose metabolism (Mao and Zhang, 2018), and thus, lower AMPK activation as well as GLUT4 and CPT1 protein content are associated with lack of mTOR signaling, and these data suggest the lack of CPT1 increases fatty acids accumulation and this effect along with GLUT4 deficiency by EtOH accelerate metabolic disorder for glucose uptake. In this context, TG accumulation induced by chronic alcohol consumption (Figure 3A) and the down-regulations of AMPK, GLUT4, CPT1, and mTOR are more notable aspects of ethanol toxicity (Figures 2B–G). Excessive levels of fatty acid or metabolites of fatty acids in muscle induced by high-fat diet have been reported to increases the incidence of insulin resistance in affected tissues (Koh et al., 2019b). Paradoxically, lipotoxicity does not induce metabolic dysregulation or insulin resistance when skeletal muscle can completely oxidize fatty acids (e.g., exercise-trained muscle). We showed that ethanol facilitates the release of fatty acid from adipose tissue and lowers the LCAD of skeletal muscle (Figure 4I) without dysfunction of mitochondrial respiration with fatty acid (Figures 4E,F). These effects by EtOH may incompletely break down of fatty acid and thus accelerated the accumulation of TG in skeletal muscle (Figure 3A), these effects are linked to insulin resistance in muscle (Kelley and Goodpaster, 2001; Gavin et al., 2018), and eventually, reduced blood glucose disposal rates (Figure 3B), because of ∼80% of glucose is taken up by skeletal muscle in the postprandial state (Thiebaud et al., 1982). It is well known that PPAR*δ* activation has shown to increase fatty acid oxidation (Fan et al., 2017), this effect involved a decrease in accumulated TG in skeletal muscle due to EtOH consumption (Figure 3A). In this context, we found PPAR*δ* increases AMPK activation (Figure 4J) and CACT as well as restores in a decreased LCAD protein content by EtOH in skeletal muscle (Figure 4I), indicating that PPAR*δ* facilitates fatty acid oxidation through accelerated Acyl-carnitine translocation into the mitochondrial matrix for bioenergetic activation via LCAD and CACT.

Alcohol consumption appears to reduce insulin sensitivity in skeletal muscle in numerous ways. A chronic high fat diet decreased insulin sensitivity in skeletal muscle in mice by typically reducing the serine and threonine phosphorylation of AKT (Koh et al., 2019b), but alcohol consumption only reduced insulin sensitivity of AKT serine 473 phosphorylation (Figures 3F,G), and this would have affected the lower glucose disposal rate in alcohol consumed mice (Figure 3B). It is well known that loss of mTORC2 (rapamycin-insensitive protein complex formed by serine/threonine kinase mTOR) signaling decreases insulin sensitivity by reducing S473-AKT phosphorylation (Lamming et al., 2012), and that Rictor (a subunit of mTORC2) deficient muscle has lower glucose uptake (Kleinert et al., 2014). In myotubes, exposure to a low amount of EtOH (30 mM) reduced the activations of Rictor and Raptor at 6 h, which reverted to normal levels at 48h (Figures 2F,G). However, frequent consumption of even relatively small amounts of alcohol may block the reestablishment of the normal mTORC2 and S473-AKT activation levels required for insulin signaling. However, we found PPAR*δ* accelerated the normalization of Rictor (Figure 3I) and insulin-induced S473-AKT activation that is decreased by EtOH (Figures 3D,E), but PPAR*δ* rather blocked the normalization of Raptor activation in EtOH exposed-myotubes (Figure 3J). These results indicate that PPAR*δ* can protect muscle that is impaired insulin sensitivity via AMPK and mTORC2 (Rictor), but not mTORC1. AMPK activation has been shown to enhance the phosphorylation of AKT at serine and threonine (Koh et al., 2019b).

It has been well established that increased release of ROS from mitochondria is a critical source of insulin resistance (Anderson et al., 2009). In the present study, although chronic alcohol consumption did not negatively influence catalase (Figure 5B), H_2_O_2_ emission from mitochondria was increased by ethanol (Figure 5C), presumably because reductase did not upregulate sufficiently to take reduce H_2_O_2_ into water and oxygen. In this context, it was shown in a previous study that endurance exercise increases fatty acid oxidation and SOD and catalase via PPAR*δ* (Fan et al., 2017), and we found PPAR*δ* activation can prevent ethanol-induced lipotoxicity by enhancing mitochondrial uncoupling with fatty acid without increasing mitochondrial ROS emission. Previous studies have shown mild uncoupling is an antioxidant factor (Mailloux and Harper, 2011), that loss of UCP3 is associated with insulin resistance, and that the restoration of UCP3 protein content improves insulin sensitivity (Mensink et al., 2007). Moreover, UCP3 overexpression in skeletal muscle lowers ROS release from mitochondria (Nabben et al., 2008; Toime and Brand, 2010). In the present study, PPAR*δ* activation decreased mitochondrial coupling ratio (Figure 4H) induced by UCP3 (Figure 4I). Summarizing, increased fatty acid oxidation induces ROS production from mitochondria; however, we showed that PPAR*δ* accelerates fatty acid utilization without inducing ROS production via mitochondrial uncoupling and that these effects prevent the development of alcohol-induced insulin resistance in skeletal muscles and whole body (Figure 3).

## Conclusion

In conclusion, the present study suggested chronic alcohol consumption induces metabolic dysregulation by accumulation of TG from adipose tissue and reducing the activations of AMPK, mTORC2, and ROS. On the other hand, PPAR*δ* recovered AMPK, mTORC2, and ROS and these effects improve alcohol induced-metabolic dysregulation associated oxidative stress by enhancing fatty acid oxidation, mitochondrial uncoupling, and antioxidant defense via UCP3. These identified functions of PPAR*δ* activation provide a number of therapeutic targets for improving insulin resistance and oxidative stress mediated by alcohol consumption.

## Data Availability Statement

All datasets generated for this study are included in the article/Supplementary Material.

## Ethics Statement

All institutional and governmental regulations regarding the Ethical use were complied with during this study, which was approved by the Institutional Animal Care and Use Committee of Yeungnam University (YUMC-AEC2018-034).

## Author Contributions

J-YK designed and supervised the study, analysis, and data interpretation. J-HK conducted the cell line, animal studies and molecular phenotyping studies, glucose uptake, IPGT test, and oxygen consumption analysis. K-HK performed the animal studies for adipose tissue. S-YP performed the cell and tissue analysis. J-HK conducted the statistical analyses. J-YK, Y-WK, and J-HK drafted the manuscript. All authors contributed to the final version of the manuscript.

## Conflict of Interest

The authors declare that the research was conducted in the absence of any commercial or financial relationships that could be construed as a potential conflict of interest.

## Supplementary Material

FIGURE S1 HSL activity in a time-dependent manner by EtOH. HSL activity was determined from adipose tissue in rats. Values are means ± SE. Significance was determined by one-way ANOVA with Tukey’s collection. **P* < 0.05 versus 0.

## References

[B1] Anderson E. J. Lustig M. E. Boyle K. E. Woodlief T. L. Kane D. A. Lin C. T. (2009). Mitochondrial H2O2 emission and cellular redox state link excess fat intake to insulin resistance in both rodents and humans. *J. Clin. Invest.* 119 573–581. 10.1172/jci37048 19188683 PMC2648700

[B2] Denton R. M. Randle P. J. (1967). Concentrations of glycerides and phospholipids in rat heart and gastrocnemius muscles. Effects of alloxandiabetes and perfusion. *Biochem. J.* 104, 416–422. 10.1042/bj1040416 6048783 PMC1270602

[B3] Dressel U. Allen T. L. Pippal J. B. Rohde P. R. Lau P. Muscat G. E. (2003). The peroxisome proliferator-activated receptor beta/delta agonist, GW501516, regulates the expression of genes involved in lipid catabolism and energy uncoupling in skeletal muscle cells. *Mol. Endocrinol.* 17 2477–2493. 10.1210/me.2003-0151 14525954

[B4] Fan W. Waizenegger W. Lin C. S. Sorrentino V. He M. X. Wall C. E. (2017). PPARdelta promotes running endurance by preserving glucose. *Cell Metab.* 25 1186–1193.e7. 10.1016/j.cmet.2017.04.006 28467934 PMC5492977

[B5] Finlin B. S. Memetimin H. Confides A. L. Kasza I. Zhu B. Vekaria H. J. (2018). Human adipose beiging in response to cold and mirabegron. *JCI Insight* 3:e121510. 10.1172/jci.insight.121510 30089732 PMC6129119

[B6] Folch J. Lees M. Sloane Stanley G. H. (1957). A simple method for the isolation and purification of total lipides from animal tissues. *J. Biol. Chem.* 226, 497–509. 13428781

[B7] Frayn K. N. Maycock P. F. (1980). Skeletal muscle triacylglycerol in the rat: methods for sampling and measurement, and studies of biological variability. *J. Lipid Res.* 21, 139–144. 7354251

[B8] Gan Z. Burkart-Hartman E. M. Han D. H. Finck B. Leone T. C. Smith E. Y. (2011). The nuclear receptor PPARbeta/delta programs muscle glucose metabolism in cooperation with AMPK and MEF2. *Genes Dev.* 25 2619–2630. 10.1101/gad.178434.111 22135324 PMC3248683

[B9] Gao B. Bataller R. (2011). Alcoholic liver disease: pathogenesis and new therapeutic targets. *Gastroenterology* 141 1572–1585. 10.1053/j.gastro.2011.09.002 21920463 PMC3214974

[B10] Gaudel C. Grimaldi P. A. (2007). Metabolic functions of peroxisome proliferator-activated receptor beta/delta in skeletal muscle. *PPAR Res.* 2007:86394. 10.1155/2007/86394 17389772 PMC1783743

[B11] Gavin T. P. Ernst J. M. Kwak H. B. Caudill S. E. Reed M. A. Garner R. T. (2018). High incomplete skeletal muscle fatty acid oxidation explains low muscle insulin sensitivity in poorly controlled T2D. *J. Clin. Endocrinol. Metab.* 103 882–889. 10.1210/jc.2017-01727 29155999

[B12] Kelley D. E. Goodpaster B. H. (2001). Skeletal muscle triglyceride. An aspect of regional adiposity and insulin resistance. *Diabetes Care* 24 933–941. 10.2337/diacare.24.5.933 11347757

[B13] Kleinert M. Sylow L. Fazakerley D. J. Krycer J. R. Thomas K. C. Oxbøll A.-J. (2014). Acute mTOR inhibition induces insulin resistance and alters substrate utilization in vivo. *Mol. Metab.* 3 630–641. 10.1016/j.molmet.2014.06.004 25161886 PMC4142396

[B14] Kocha T. Yamaguchi M. Ohtaki H. Fukuda T. Aoyagi T. (1997). Hydrogen peroxide-mediated degradation of protein: different oxidation modes of copper- and iron-dependent hydroxyl radicals on the degradation of albumin. *Biochim. Biophys. Acta* 1337 319–326. 10.1016/s0167-4838(96)00180-x 9048910

[B15] Koh J. H. Hancock C. R. Han D. H. Holloszy J. O. Nair K. S. Dasari S. (2019a). AMPK and PPARbeta positive feedback loop regulates endurance exercise training-mediated GLUT4 expression in skeletal muscle. *Am. J. Physiol. Endocrinol. Metab.* 316 E931–E939. 10.1152/ajpendo.00460.2018 30888859 PMC6580175

[B16] Koh J. H. Hancock C. R. Terada S. Higashida K. Holloszy J. O. Han D. H. (2017). PPARbeta is essential for maintaining normal levels of PGC-1alpha and mitochondria and for the increase in muscle mitochondria induced by exercise. *Cell Metab.* 25 1176–1185.e5. 10.1016/j.cmet.2017.04.029 28467933 PMC5894349

[B17] Koh J. H. Johnson M. L. Dasari S. LeBrasseur N. K. Vuckovic I. Henderson G. C. (2019b). TFAM enhances fat oxidation and attenuates high-fat diet-induced insulin resistance in skeletal muscle. *Diabetes* 68 1552–1564. 10.2337/db19-0088 31088855 PMC6692815

[B18] Lamming D. W. Ye L. Katajisto P. Goncalves M. D. Saitoh M. Stevens D. M. (2012). Rapamycin-induced insulin resistance is mediated by mTORC2 loss and uncoupled from longevity. *Science* 335 1638–1643. 10.1126/science.1215135 22461615 PMC3324089

[B19] Lea W. Abbas A. S. Sprecher H. Vockley J. Schulz H. (2000). Long-chain acyl-CoA dehydrogenase is a key enzyme in the mitochondrial beta-oxidation of unsaturated fatty acids. *Biochim. Biophys. Acta* 1485 121–128. 10.1016/s1388-1981(00)00034-2 10832093

[B20] Louvet A. Mathurin P. (2015). Alcoholic liver disease: mechanisms of injury and targeted treatment. *Nat. Rev. Gastroenterol. Hepatol.* 12 231–242. 10.1038/nrgastro.2015.35 25782093

[B21] Mailloux R. J. Harper M. E. (2011). Uncoupling proteins and the control of mitochondrial reactive oxygen species production. *Free Radic. Biol. Med.* 51 1106–1115. 10.1016/j.freeradbiomed.2011.06.022 21762777

[B22] Mao Z. Zhang W. (2018). Role of mTOR in glucose and lipid metabolism. *Int. J. Mol. Sci.* 19:2043. 10.3390/ijms19072043 30011848 PMC6073766

[B23] Mensink M. Hesselink M. K. Borghouts L. B. Keizer H. Moonen-Kornips E. Schaart G. (2007). Skeletal muscle uncoupling protein-3 restores upon intervention in the prediabetic and diabetic state: implications for diabetes pathogenesis? *Diabetes Obes Metab.* 9 594–596. 10.1111/j.1463-1326.2006.00628.x 17587402

[B24] Nabben M. Hoeks J. Briede J. J. Glatz J. F. Moonen-Kornips E. Hesselink M. K. (2008). The effect of UCP3 overexpression on mitochondrial ROS production in skeletal muscle of young versus aged mice. *FEBS Lett.* 582 4147–4152. 10.1016/j.febslet.2008.11.016 19041310

[B25] Ogawa A. Yamamoto S. Kanazawa M. Takayanagi M. Hasegawa S. Kohno Y. (2000). Identification of two novel mutations of the carnitine/acylcarnitine translocase (CACT) gene in a patient with CACT deficiency. *J. Hum. Genet.* 45 52–55. 10.1007/s100380050010 10697964

[B26] O’Neill H. M. Holloway G. P. Steinberg G. R. (2013). AMPK regulation of fatty acid metabolism and mitochondrial biogenesis: implications for obesity. *Mol. Cell Endocrinol.* 366 135–151. 10.1016/j.mce.2012.06.019 22750049

[B27] Pande S. V. Brivet M. Slama A. Demaugre F. Aufrant C. Saudubray J. M. (1993). Carnitine-acylcarnitine translocase deficiency with severe hypoglycemia and auriculo ventricular block. Translocase assay in permeabilized fibroblasts. *J. Clin. Invest.* 91 1247–1252. 10.1172/jci116288 8450053 PMC288085

[B28] Rhein V. Giese M. Baysang G. Meier F. Rao S. Schulz K. L. (2010). Ginkgo biloba extract ameliorates oxidative phosphorylation performance and rescues abeta-induced failure. *PLoS One* 5:e12359. 10.1371/journal.pone.0012359 20808761 PMC2927422

[B29] Shin M. G. Cha H. N. Park S. Kim Y. W. Kim J. Y. Park S. Y. (2019). Selenoprotein W deficiency does not affect oxidative stress and insulin sensitivity in the skeletal muscle of high-fat diet-fed obese mice. *Am. J. Physiol. Cell Physiol.* 317 C1172–C1182. 10.1152/ajpcell.00064.2019 31509445

[B30] Tanaka T. Yamamoto J. Iwasaki S. Asaba H. Hamura H. Ikeda Y. (2003). Activation of peroxisome proliferator-activated receptor delta induces fatty acid beta-oxidation in skeletal muscle and attenuates metabolic syndrome. *Proc. Natl. Acad. Sci. U.S.A.* 100 15924–15929. 10.1073/pnas.0306981100 14676330 PMC307669

[B31] Thiebaud D. Jacot E. DeFronzo R. A. Maeder E. Jequier E. Felber J. P. (1982). The effect of graded doses of insulin on total glucose uptake, glucose oxidation, and glucose storage in man. *Diabetes* 31 957–963. 10.2337/diacare.31.11.957 6757014

[B32] Toime L. J. Brand M. D. (2010). Uncoupling protein-3 lowers reactive oxygen species production in isolated mitochondria. *Free Radic. Biol. Med.* 49 606–611. 10.1016/j.freeradbiomed.2010.05.010 20493945 PMC2903626

[B33] Wang Y. X. Lee C. H. Tiep S. Yu R. T. Ham J. Kang H. (2003). Peroxisome-proliferator-activated receptor delta activates fat metabolism to prevent obesity. *Cell* 113 159–170. 10.1016/s0092-8674(03)00269-1 12705865

[B34] Wicks S. E. Vandanmagsar B. Haynie K. R. Fuller S. E. Warfel J. D. Stephens J. M. (2015). Impaired mitochondrial fat oxidation induces adaptive remodeling of muscle metabolism. *Proc. Natl. Acad. Sci. U.S.A.* 112 E3300–E3309. 10.1073/pnas.1418560112 26056297 PMC4485116

[B35] Zhang W. Zhong W. Sun Q. Sun X. Zhou Z. (2017). Hepatic overproduction of 13-HODE due to ALOX15 upregulation contributes to alcohol-induced liver injury in mice. *Sci. Rep.* 7:8976. 10.1038/s41598-017-02759-0 28827690 PMC5567196

[B36] Zhong W. Zhao Y. Tang Y. Wei X. Shi X. Sun W. (2012). Chronic alcohol exposure stimulates adipose tissue lipolysis in mice: role of reverse triglyceride transport in the pathogenesis of alcoholic steatosis. *Am. J. Pathol.* 180 998–1007. 10.1016/j.ajpath.2011.11.017 22234172 PMC3349880

