# Supplementary figures and images for: PPARδ Attenuates Alcohol-Mediated Insulin Resistance by Enhancing Fatty Acid-Induced Mitochondrial Uncoupling and Antioxidant Defense in Skeletal Muscle
Source: Front Physiol. 2020 Jul 14;11:749. doi: 10.3389/fphys.2020.00749 (PMC7372095; doi:10.3389/fphys.2020.00749)

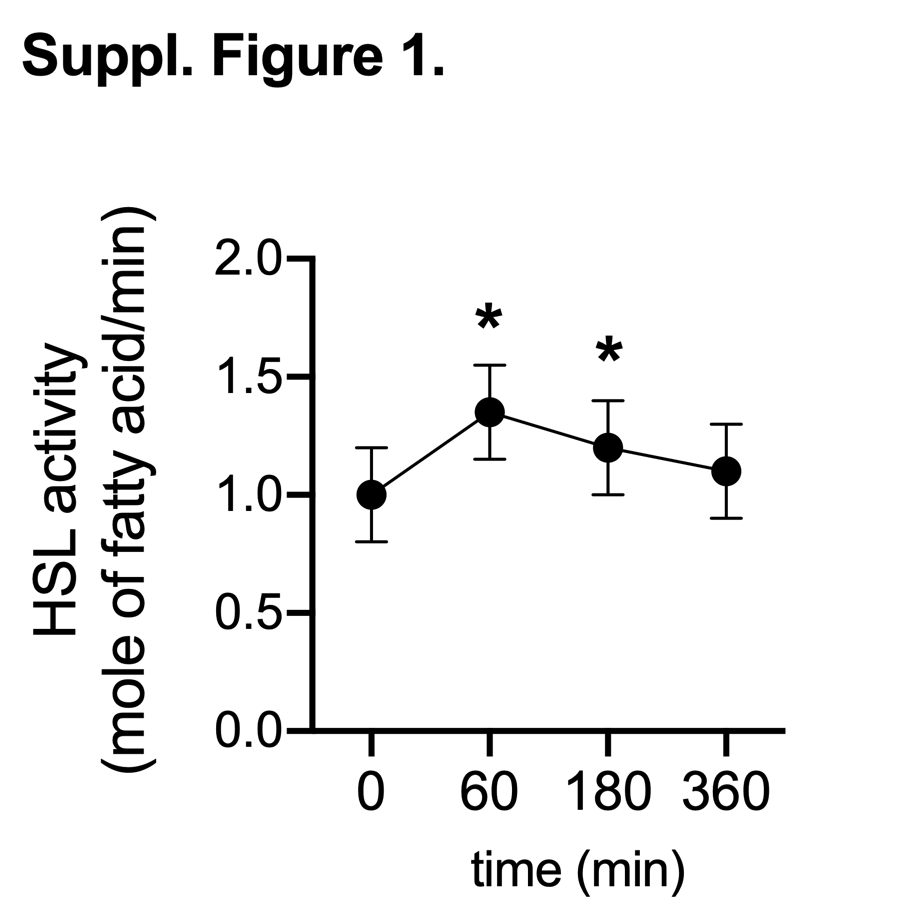

Supplement: FIGURE S1 — HSL activity in a time-dependent manner by EtOH. HSL activity was determined from adipose tissue in rats. Values are means ± SE. Significance was determined by one-way ANOVA with Tukey’s collection. *P < 0.05 versus 0. [file Image_1.tiff]
